# Supplementary material for: Tumor cell villages define the co-dependency of tumor and microenvironment in liver cancer
Source: Nat Commun. 2026 Feb 21;17:1986. doi: 10.1038/s41467-026-69797-z (PMC12932787; doi:10.1038/s41467-026-69797-z)
Supplement: Supplementary file 1 — Supplementary Information [file 41467_2026_69797_MOESM1_ESM.pdf]

Supplementary information

Tumor cell villages define the co-dependency of tumor and microenvironment in liver cancer

Meng Liu, Maria O. Hernandez, Darko Castven, Hsin-Pei Lee, Wenqi Wu, Limin Wang, Marshonna Forgues, Jonathan M. Hernandez, Jens U. Marquardt, Lichun Ma

This file contains:
Supplementary Figures 1-12

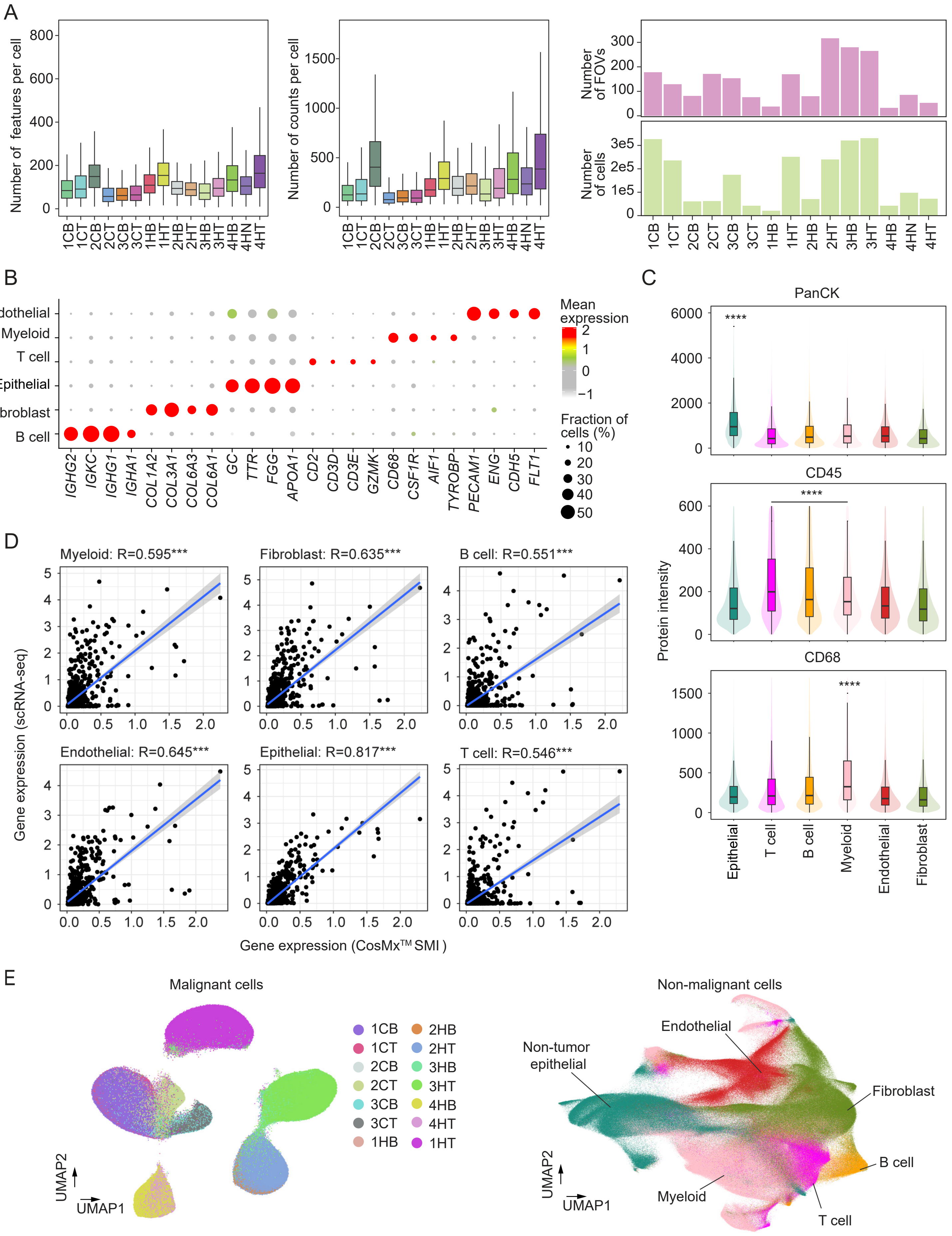
A
Number of features per cell
Number of counts per cell
Number of FOVs
Number of cells
1CB
1CT
2CB
2CT
3CB
3CT
1HB
1HT
2HB
2HT
3HB
3HT
4HB
4HN
4HT
B
Endothelial
Myeloid
T cell
Epithelial
Fibroblast
B cell
IGHG2
IGKC
IGHG1
IGHA1
COL1A2
COL3A1
COL6A3
COL6A1
GC
TTR
FGG
APOA1
CD2
CD3D
CD3E
GZMK
CD68
CSF1R
AIF1
TYROBP
PECAM1
ENG
CDH5
FLT1
Mean expression
Fraction of cells (%)
C
PanCK
CD45
CD68
Protein intensity
Epithelial
T cell
B cell
Myeloid
Endothelial
Fibroblast
D
Myeloid: R=0.595***
Fibroblast: R=0.635***
B cell: R=0.551***
Endothelial: R=0.645***
Epithelial: R=0.817***
T cell: R=0.546***
Gene expression (scRNA-seq)
Gene expression (CosMx™ SMI)
E
Malignant cells
Non-malignant cells
1CB
1CT
2CB
2CT
3CB
3CT
1HB
2HB
2HT
3HB
3HT
4HB
4HT
1HT
UMAP1
UMAP2
Endothelial
Non-tumor epithelial
Fibroblast
B cell
Myeloid
T cell

Supplementary Fig. 1: Quality control of the CosMx^{TM} SMI data.

(A) Boxplots of the number of genes or counts detected per cell in each sample (left two panels). Each box shows the median (center line), interquartile range (box), and data range (whiskers). Bar plots of the number of field of views (FOVs) and the number of cells in each sample (right panel). Sample IDs were named based on histological subtypes of liver cancer, where H represents HCC and C represents iCCA. Source data are provided as a Source Data file.

(B) Expression of cell type-specific marker genes. Color indicates normalized expression and dot size indicates the fraction of cells expressing a certain gene.

(C) Protein intensities of Pan-CK (Pan-Cytokeratin), CD45, and CD68 in each cell type. Each box shows the median (center line), interquartile range (box), and data range (whiskers). P-values were calculated with one-sided Student's t-test by comparing the markers of the corresponding cell type and all other cells (Pan-CK for epithelial cells, CD45 for immune cells, and CD68 for myeloid cells). ****, p-value <0.0001. Source data are provided as a Source Data file.

(D) Correlation of gene expression between CosMx^{TM} SMI data and scRNA-seq data in individual cell types. Each dot represents a gene. Pearson correlation coefficients and p-values are shown for each cell type. ***, p-value < 0.001. Linear regression was applied. Source data are provided as a Source Data file.

(E) UMAP embeddings of malignant cells (left, colored by samples) and non-malignant cells (right, colored by cell types). Sample IDs were named based on histological subtypes of liver cancer, where H represents HCC and C represents iCCA.

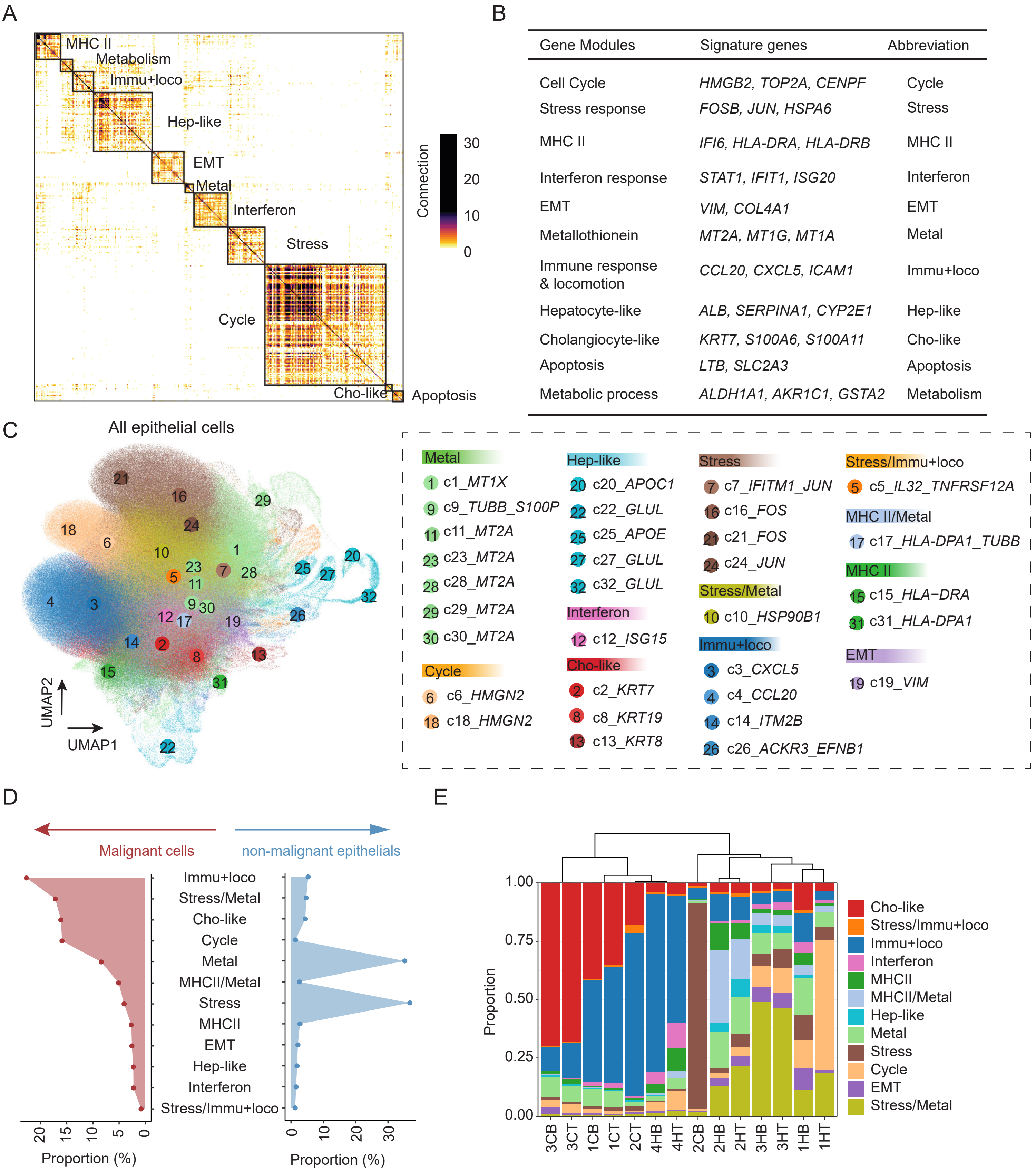

A
MHC II
Metabolism
Immu+loco
Hep-like
EMT
Metal
Interferon
Stress
Cycle
Cho-like
Apoptosis
Connection
30
20
10
0
B
Gene Modules | Signature genes | Abbreviation
Cell Cycle | HMGB2, TOP2A, CENPF | Cycle
Stress response | FOSB, JUN, HSPA6 | Stress
MHC II | IFI6, HLA-DRA, HLA-DRB | MHC II
Interferon response | STAT1, IFIT1, ISG20 | Interferon
EMT | VIM, COL4A1 | EMT
Metallothionein | MT2A, MT1G, MT1A | Metal
Immune response & locomotion | CCL20, CXCL5, ICAM1 | Immu+loco
Hepatocyte-like | ALB, SERPINA1, CYP2E1 | Hep-like
Cholangiocyte-like | KRT7, S100A6, S100A11 | Cho-like
Apoptosis | LTB, SLC2A3 | Apoptosis
Metabolic process | ALDH1A1, AKR1C1, GSTA2 | Metabolism
C
All epithelial cells
UMAP1
UMAP2
Metal
1 c1_MT1X
9 c9_TUBB_S100P
11 c11_MT2A
23 c23_MT2A
28 c28_MT2A
29 c29_MT2A
30 c30_MT2A
Cycle
6 c6_HMGN2
18 c18_HMGN2
Hep-like
20 c20_APOC1
22 c22_GLUL
25 c25_APOE
27 c27_GLUL
32 c32_GLUL
Interferon
12 c12_ISG15
Cho-like
2 c2_KRT7
8 c8_KRT19
13 c13_KRT8
Stress
7 c7_IFITM1_JUN
16 c16_FOS
21 c21_FOS
24 c24_JUN
Stress/Metal
10 c10_HSP90B1
Immu+loco
3 c3_CXCL5
4 c4_CCL20
14 c14_ITM2B
26 c26_ACKR3_EFNB1
Stress/Immu+loco
5 c5_IL32_TNFRSF12A
MHC II/Metal
17 c17_HLA-DPA1_TUBB
MHC II
15 c15_HLA-DRA
31 c31_HLA-DPA1
EMT
19 c19_VIM
D
Malignant cells
non-malignant epithelials
Immu+loco
Stress/Metal
Cho-like
Cycle
Metal
MHCII/Metal
Stress
MHCII
EMT
Hep-like
Interferon
Stress/Immu+loco
Proportion (%)
20 15 10 5 0
0 10 20 30
Proportion (%)
E
Proportion
1.00
0.75
0.50
0.25
0.00
3CB 3CT 1CB 1CT 2CT 4HB 4HT 2CB 2HB 2HT 3HB 3HT 1HB 1HT
Cho-like
Stress/Immu+loco
Immu+loco
Interferon
MHCII
MHCII/Metal
Hep-like
Metal
Stress
Cycle
EMT
Stress/Metal

Supplementary Fig. 2: Transcriptomic states of epithelial cells in liver cancer.

(A) Heatmap of module-specific genes. Connections of genes were calculated as the number of individual tumor modules in which they co-occurred (see **Methods** for details).

(B) Recurrent genes modules, key representative genes, and abbreviations of the identified gene modules.

(C) UMAP embeddings of all epithelial cells (including malignant cells and non-malignant epithelial cells) from the CosMx™ SMI data based on gene module scores. Clusters were annotated based on module scores and marker genes.

(D) Proportion of each transcriptomic state in malignant cells (left) and non-malignant epithelial cells (right).

(E) Composition of malignant cell transcriptomic states in each tumor sample. Sample IDs were named based on histological subtypes of liver cancer, where H represents HCC and C represents iCCA.

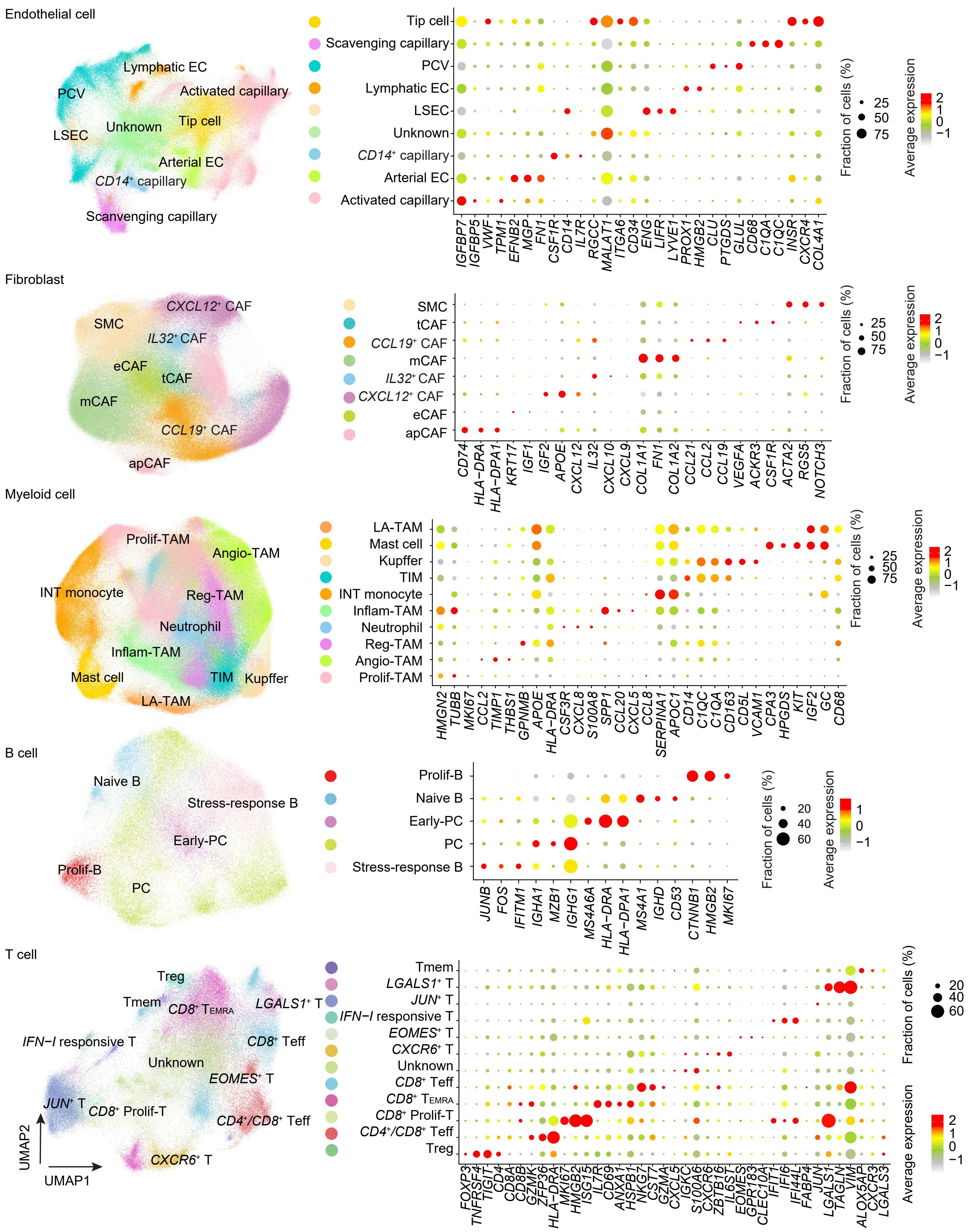

Endothelial cell
Lymphatic EC
PCV
Activated capillary
Tip cell
LSEC
Unknown
Arterial EC
CD14+ capillary
Scanvenging capillary
Tip cell
Scavenging capillary
PCV
Lymphatic EC
LSEC
Unknown
CD14+ capillary
Arterial EC
Activated capillary
IGFBP7 IGFBP5 VWF TPM1 EFNB2 MGP FN1 CSF1R CD14 IL7R RGCC MALAT1 ITGA6 CD34 ENG LIFR LYVE1 PROX1 HMGB2 CLU PTGDS GLUL CD68 C1QA C1QC INSR CXCR4 COL4A1
Fraction of cells (%)
25
50
75
Average expression
2
1
0
−1
Fibroblast
CXCL12+ CAF
SMC
IL32+ CAF
eCAF
tCAF
mCAF
CCL19+ CAF
apCAF
SMC
tCAF
CCL19+ CAF
mCAF
IL32+ CAF
CXCL12+ CAF
eCAF
apCAF
CD74 HLA−DRA HLA−DPA1 KRT17 IGF1 IGF2 APOE CXCL12 IL32 CXCL10 CXCL9 COL1A1 FN1 COL1A2 CCL21 CCL2 CCL19 VEGFA ACKR3 CSF1R ACTA2 RGS5 NOTCH3
Myeloid cell
Prolif-TAM
Angio-TAM
INT monocyte
Reg-TAM
Neutrophil
Inflam-TAM
Mast cell
TIM
Kupffer
LA-TAM
LA-TAM
Mast cell
Kupffer
TIM
INT monocyte
Inflam-TAM
Neutrophil
Reg-TAM
Angio-TAM
Prolif-TAM
HMGN2 TUBB MKI67 CCL2 TIMP1 THBS1 GPNMB APOE HLA−DRA CSF3R CXCL8 S100A8 SPP1 CCL20 CXCL5 CCL8 SERPINA1 APOC1 CD14 C1QC C1QA CD163 CD5L VCAM1 CPA3 HPGDS KIT IGF2 GC CD68
B cell
Naive B
Stress-response B
Early-PC
Prolif-B
PC
Prolif-B
Naive B
Early-PC
PC
Stress-response B
JUNB FOS IFITM1 IGHA1 MZB1 IGHG1 MS4A6A HLA−DRA HLA−DPA1 MS4A1 IGHD CD53 CTNNB1 HMGB2 MKI67
Fraction of cells (%)
20
40
60
Average expression
1
0
−1
T cell
Treg
Tmem
CD8+ TEMRA
LGALS1+ T
IFN−I responsive T
CD8+ Teff
Unknown
EOMES+ T
JUN+ T
CD8+ Prolif-T
CD4+/CD8+ Teff
CXCR6+ T
UMAP2
UMAP1
Tmem
LGALS1+ T
JUN+ T
IFN−I responsive T
EOMES+ T
CXCR6+ T
Unknown
CD8+ Teff
CD8+ TEMRA
CD8+ Prolif-T
CD4+/CD8+ Teff
Treg
FOXP3 TNFRSF4 TIGIT CD4 CD8A CD8B GZMK ZFP36 HLA−DRA MKI67 HMGB2 ISG15 IL7R CD69 ANXA1 HSPB1 NKG7 CST7 GZMA CXCL5 IGKC S100A6 CXCR6 ZBTB16 IL6ST EOMES GPR183 CLEC10A IFIT1 IFI6 IFI44L FABP4 JUN LGALS1 TAGLN VIM ALOX5AP CXCR3 LGALS3
Fraction of cells (%)
20
40
60
Average expression
2
1
0
−1

Supplementary Fig. 3: Landscape of non-malignant cells.

UMAP of each non-malignant cell type (left) and the expression of marker genes (right). In each UMAP, cell subtypes are indicated by colors. In the dot plots, color indicates normalized average gene expression, and the size of the dots stand for the fraction of cells expressing a certain gene. EC, endothelial cell PCV; post-capillary venule; LSEC, liver sinusoidal endothelial cell; CAF, cancer-associated fibroblast; mCAF, matrix CAF; eCAF, EMT-like CAF; SMC, smooth muscle cell; tCAF, tumour-like CAF; apCAF, antigen-presenting CAF; TAM, tumor-associated macrophage; Prolif-TAM, proliferating TAM; TIM, tumor infiltrating monocyte; Reg-TAM, immune-regulatory TAM; Angio-TAM, pro-angiogenic TAM; INT monocyte, intermediate monocyte; LA-TAM, lipid-associated TAM; Inflam-TAM, inflammatory cytokine-enriched TAM; PC, plasma cell; Prolif-B, proliferative B cell; Teff, effector T cell; Tmem, Memory T cell; TEMRA, recently activated effector memory or effector T cell; Treg, regulatory T cell; Prolif-T, proliferative T cell.

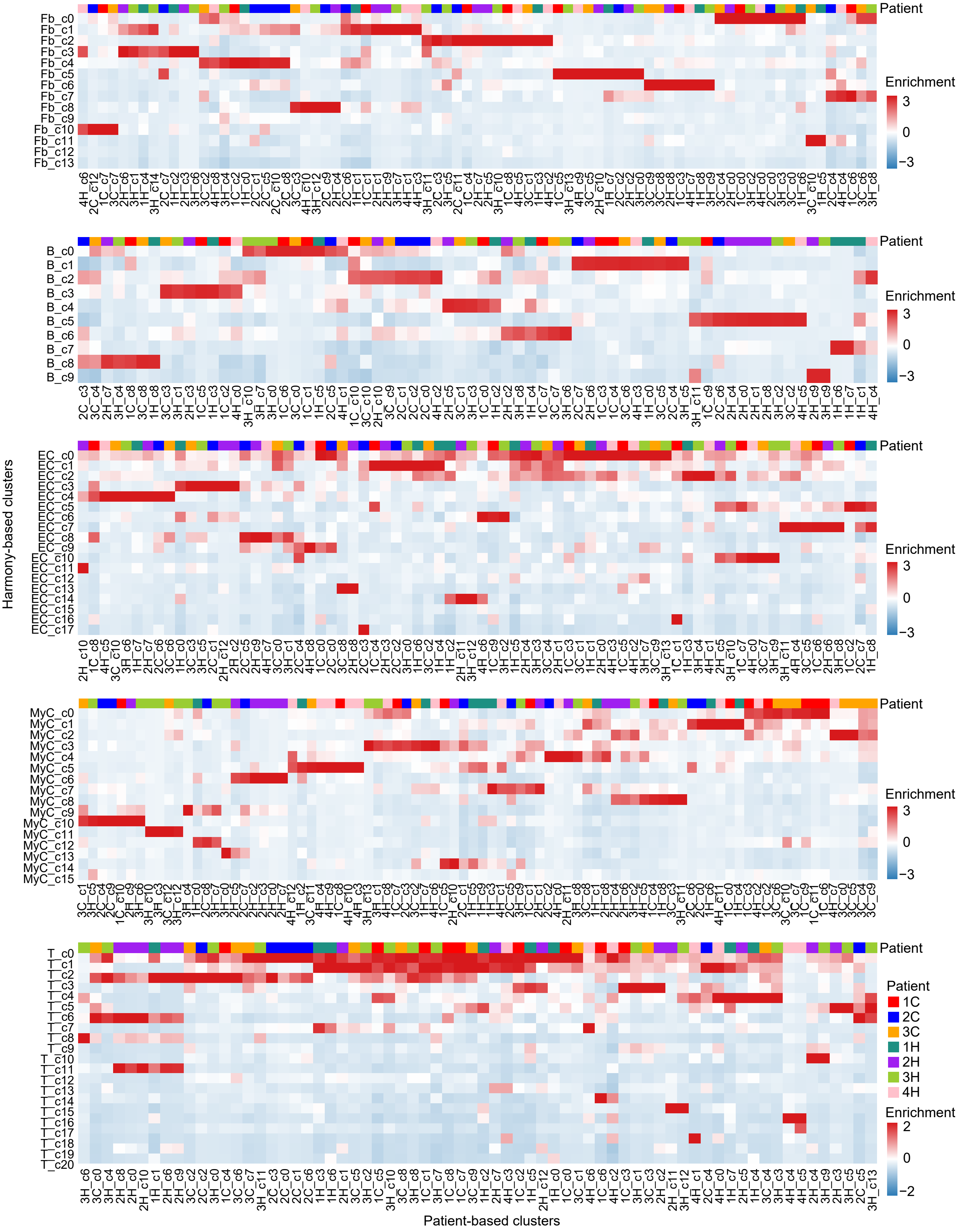
Patient
Enrichment
Fb_c0
Fb_c1
Fb_c2
Fb_c3
Fb_c4
Fb_c5
Fb_c6
Fb_c7
Fb_c8
Fb_c9
Fb_c10
Fb_c11
Fb_c12
Fb_c13
B_c0
B_c1
B_c2
B_c3
B_c4
B_c5
B_c6
B_c7
B_c8
B_c9
EC_c0
EC_c1
EC_c2
EC_c3
EC_c4
EC_c5
EC_c6
EC_c7
EC_c8
EC_c9
EC_c10
EC_c11
EC_c12
EC_c13
EC_c14
EC_c15
EC_c16
EC_c17
MyC_c0
MyC_c1
MyC_c2
MyC_c3
MyC_c4
MyC_c5
MyC_c6
MyC_c7
MyC_c8
MyC_c9
MyC_c10
MyC_c11
MyC_c12
MyC_c13
MyC_c14
MyC_c15
T_c0
T_c1
T_c2
T_c3
T_c4
T_c5
T_c6
T_c7
T_c8
T_c9
T_c10
T_c11
T_c12
T_c13
T_c14
T_c15
T_c16
T_c17
T_c18
T_c19
T_c20
3
0
−3
2
−2
1C
2C
3C
1H
2H
3H
4H
Harmony-based clusters
Patient-based clusters

Supplementary Fig. 4: Comparison between clusters identified by Harmony integration and those obtained by clustering each cell type within individual patients.

For each patient-derived cluster (column), enrichment was calculated as the scaled proportion of its cells annotated to each Harmony-derived cluster (row). The annotation bar at the top of each heatmap indicates the patients. Sample IDs were named based on histological subtypes of liver cancer, where H represents HCC and C represents iCCA. Fb, fibroblast; B, B cell; EC, endothelial cell; MyC, myeloid cell; T, T cell.

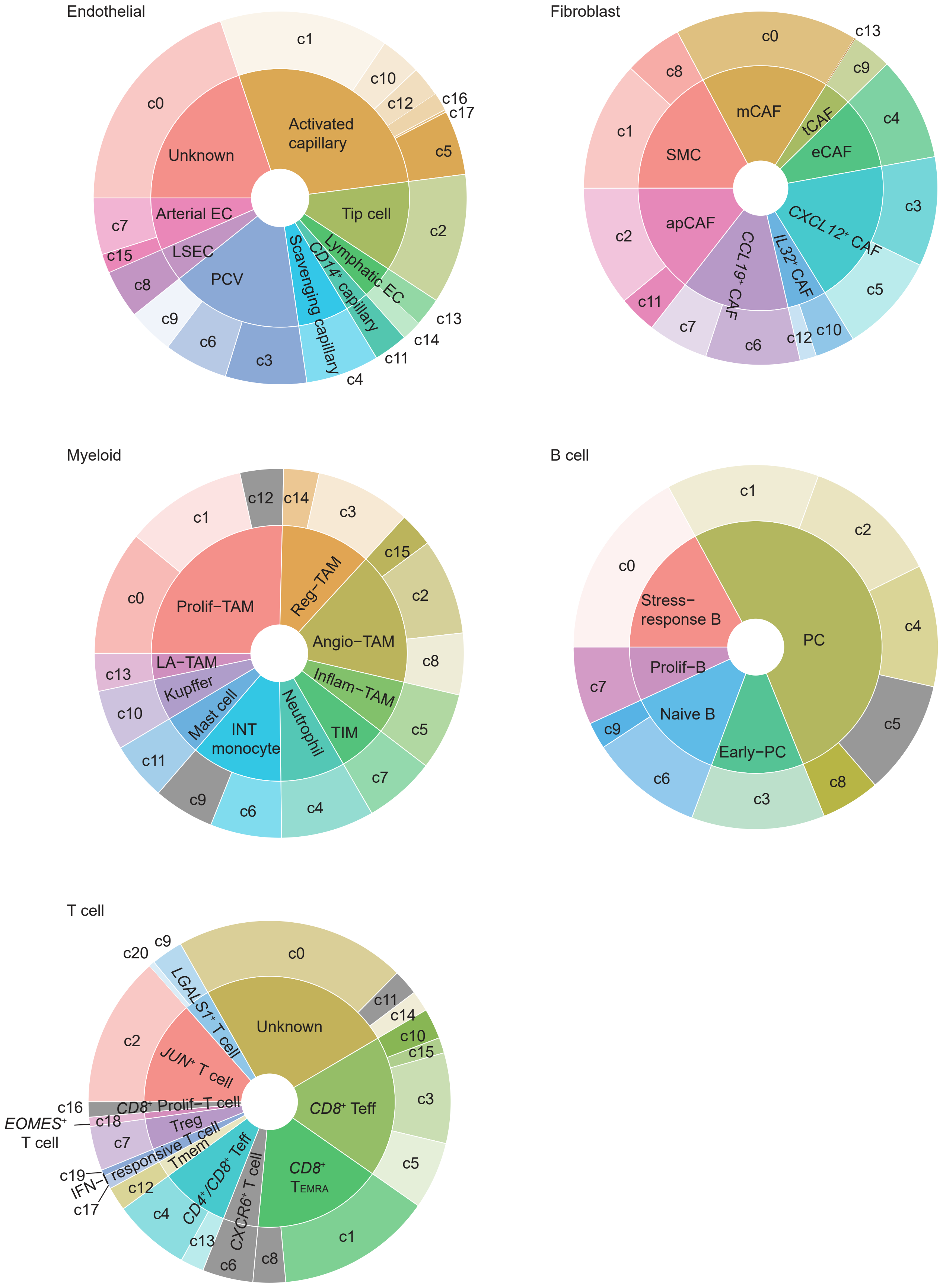
Endothelial
c1
c0
c10
c12
c16
c17
Activated capillary
Unknown
c5
Tip cell
c2
Arterial EC
c7
c15
LSEC
c8
PCV
Scavenging capillary
CD14+ capillary
Lymphatic EC
c13
c14
c11
c9
c6
c3
c4
Fibroblast
c0
c13
c8
c9
mCAF
c1
SMC
tCAF
eCAF
c4
c3
apCAF
CXCL12+ CAF
c2
CCL19+ CAF
IL32+ CAF
c5
c11
c7
c6
c12
c10
Myeloid
c12
c14
c3
c1
c15
Prolif−TAM
Reg−TAM
c0
Angio−TAM
c2
c8
LA−TAM
c13
Kupffer
Inflam−TAM
c10
Mast cell
INT monocyte
Neutrophil
TIM
c5
c11
c7
c9
c6
c4
B cell
c1
c2
c0
Stress−response B
PC
c4
Prolif−B
c7
Naive B
c9
Early−PC
c5
c6
c3
c8
T cell
c9
c20
c0
LGALS1+ T cell
c11
Unknown
c14
c10
c2
JUN+ T cell
c15
c3
c16
CD8+ Prolif−T cell
CD8+ Teff
EOMES+ T cell
c18
Treg
c7
IFN−I responsive T cell
Tmem
c5
c19
c12
CD4+/CD8+ Teff
CD8+ TEMRA
c17
c4
CXCR6+ T cell
c13
c6
c8
c1

Supplementary Fig. 5: Comparison of cell clusters identified from the CosMx™ SMI data and the scRNA-seq data from the same set of liver cancer patients.

Donut plots of cell subtypes and clusters identified from the CosMx™ SMI data. Cell subtypes or clusters found exclusively in the CosMx™ SMI data are shown in grey, while those identified in both CosMx™ SMI data and scRNA-seq data are shown in other colors. The proportion of cell subtypes or clusters in Fig. 2C from the CosMx™ SMI dataset is shown.

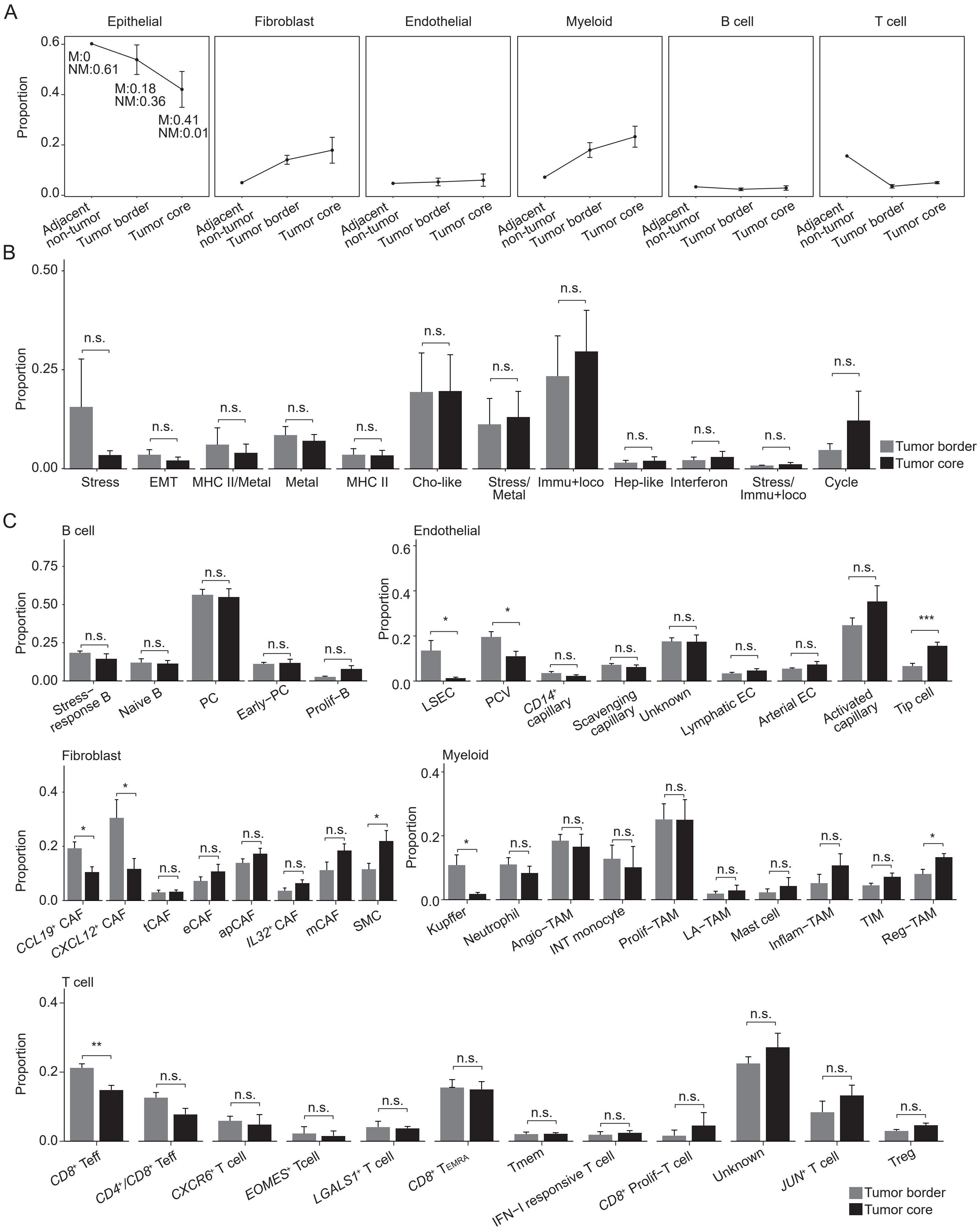
A
Epithelial
Fibroblast
Endothelial
Myeloid
B cell
T cell
Proportion
0.0
0.2
0.4
0.6
M:0
NM:0.61
M:0.18
NM:0.36
M:0.41
NM:0.01
Adjacent non-tumor
Tumor border
Tumor core
B
Proportion
0.00
0.25
0.50
n.s.
Stress
EMT
MHC II/Metal
Metal
MHC II
Cho-like
Stress/ Metal
Immu+loco
Hep-like
Interferon
Stress/ Immu+loco
Cycle
Tumor border
Tumor core
C
B cell
Proportion
0.00
0.25
0.50
0.75
Stress−response B
Naive B
PC
Early−PC
Prolif−B
Endothelial
0.0
0.2
0.4
0.6
*
***
LSEC
PCV
CD14+ capillary
Scavenging capillary
Unknown
Lymphatic EC
Arterial EC
Activated capillary
Tip cell
Fibroblast
0.0
0.2
0.4
CCL19+ CAF
CXCL12+ CAF
tCAF
eCAF
apCAF
IL32+ CAF
mCAF
SMC
Myeloid
Kupffer
Neutrophil
Angio−TAM
INT monocyte
Prolif−TAM
LA−TAM
Mast cell
Inflam−TAM
TIM
Reg−TAM
T cell
**
CD8+ Teff
CD4+/CD8+ Teff
CXCR6+ T cell
EOMES+ Tcell
LGALS1+ T cell
CD8+ TEMRA
Tmem
IFN−I responsive T cell
CD8+ Prolif−T cell
Unknown
JUN+ T cell
Treg
Tumor border
Tumor core

Supplementary Fig. 6: Enrichment of cell (sub)types across different tumor regions.

(A) Abundance of each major cell type across different tumor regions. For each cell type, the mean ± standard error of the mean (SEM) of cell fractions is shown. For epithelial cells, the total epithelial abundance was plotted, with the mean proportions of malignant (M) and non-malignant (NM) epithelial cells in each region indicated in text. Source data are provided as a Source Data file.

(B) Abundance of tumor cell states in the tumor border and tumor core. For each tumor cell state, the mean + SEM of the cell fractions is shown. p-values were calculated using two-sided Student's t-test. n.s., not significant. Source data are provided as a Source Data file.

(C) Abundance of non-malignant cell subtypes in the tumor border and tumor. For each subtype, the mean + SEM of the cell fractions is shown. p-values were calculated using two-sided Student's t-test. n.s., not significant; *, p-value<0.05; **, p-value<0.01, ***, p-value<0.001. Source data are provided as a Source Data file.

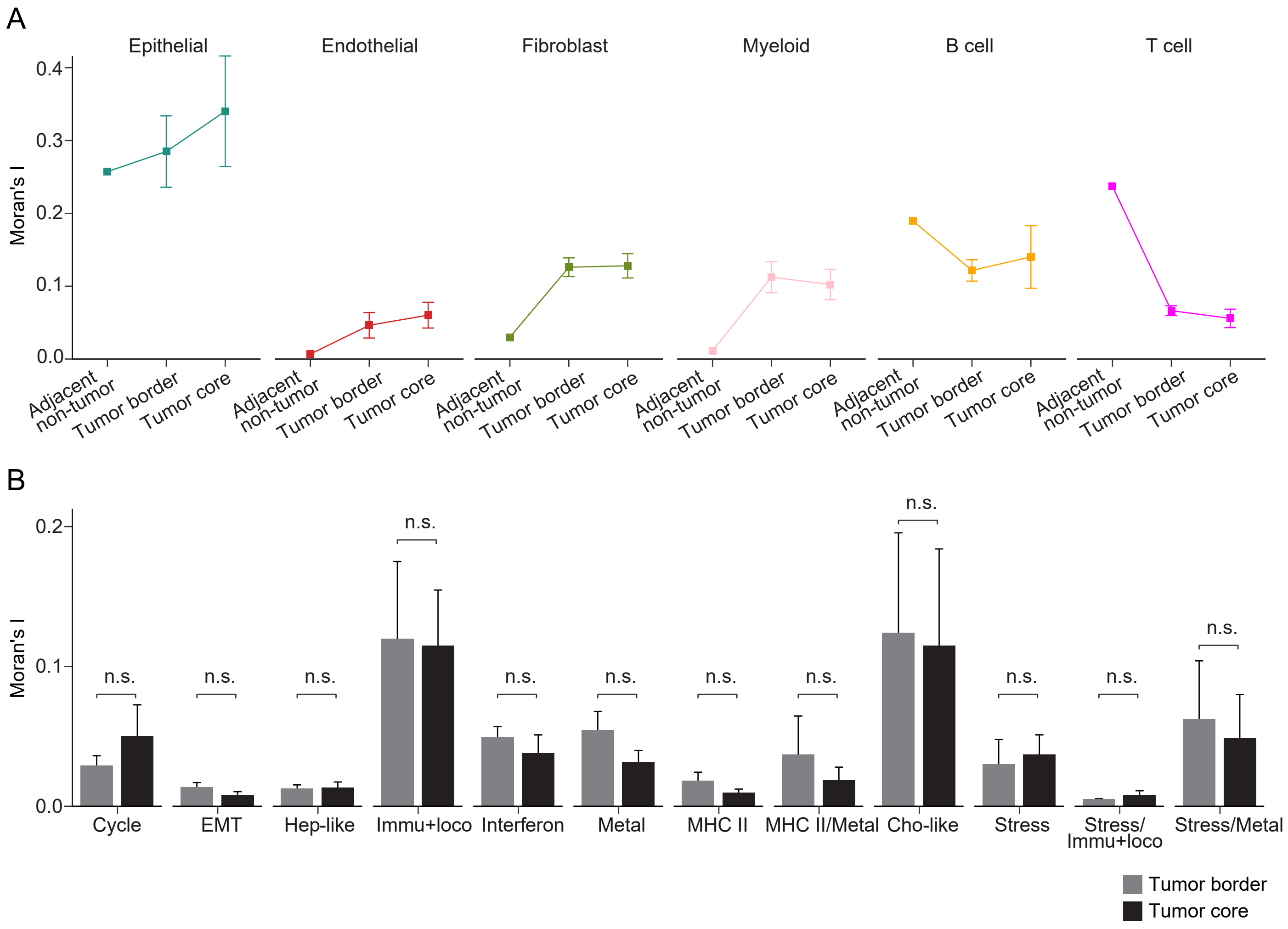

Supplementary Fig. 7: Spatial variations of cells within individual tumor regions.
(A) Global Moran's I of each major cell type within individual tumor regions. Mean ± SEM of the values are provided. Source data are provided as a Source Data file.
(B) Global Moran's I of each tumor cell state within individual tumor regions. Mean + SEM of the values are provided. p-values were calculated using two-sided Student's t-test. n.s., not significant. Source data are provided as a Source Data file.

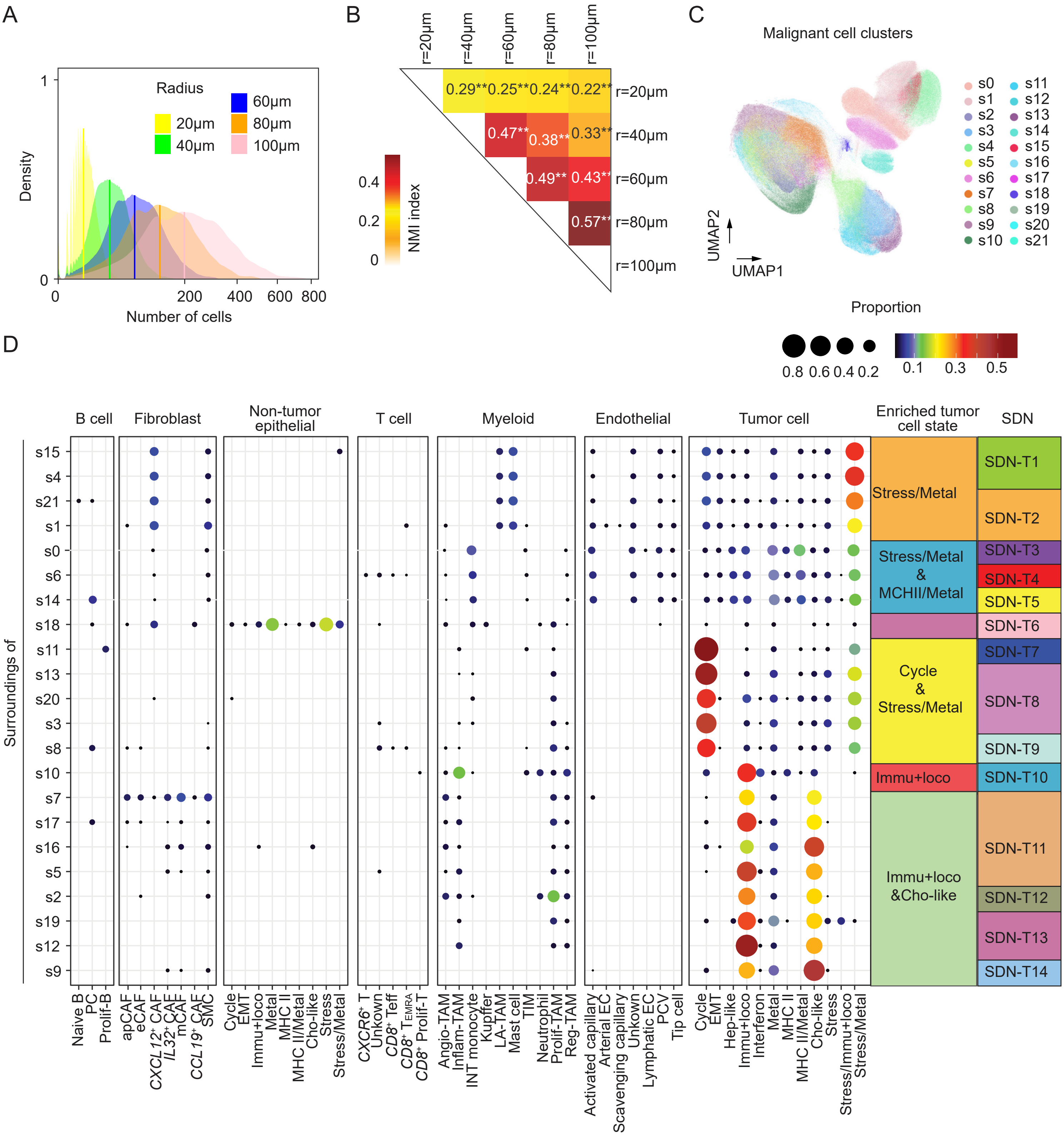
A
Radius
20µm
40µm
60µm
80µm
100µm
Density
Number of cells
B
r=20µm
r=40µm
r=60µm
r=80µm
r=100µm
0.29**
0.25**
0.24**
0.22**
0.47**
0.38**
0.33**
0.49**
0.43**
0.57**
NMI index
C
Malignant cell clusters
UMAP1
UMAP2
s0 s1 s2 s3 s4 s5 s6 s7 s8 s9 s10 s11 s12 s13 s14 s15 s16 s17 s18 s19 s20 s21
D
Proportion
B cell
Fibroblast
Non-tumor epithelial
T cell
Myeloid
Endothelial
Tumor cell
Enriched tumor cell state
SDN
Surroundings of
Stress/Metal
Stress/Metal & MCHII/Metal
Cycle & Stress/Metal
Immu+loco
Immu+loco &Cho-like
SDN-T1
SDN-T2
SDN-T3
SDN-T4
SDN-T5
SDN-T6
SDN-T7
SDN-T8
SDN-T9
SDN-T10
SDN-T11
SDN-T12
SDN-T13
SDN-T14
Naive B
PC
Prolif-B
apCAF
eCAF
CXCL12+ CAF
IL32+ CAF
mCAF
CCL19+ CAF
SMC
Cycle
EMT
Immu+loco
Metal
MHC II
MHC II/Metal
Cho-like
Stress
Stress/Metal
CXCR6+ T
Unkown
CD8+ Teff
CD8+ TEMRA
CD8+ Prolif-T
Angio-TAM
Inflam-TAM
INT monocyte
Kupffer
LA-TAM
Mast cell
TIM
Neutrophil
Prolif-TAM
Reg-TAM
Activated capillary
Arterial EC
Scavenging capillary
Unkown
Lymphatic EC
PCV
Tip cell
Cycle
EMT
Hep-like
Immu+loco
Interferon
Metal
MHC II
MHC II/Metal
Cho-like
Stress
Stress/Immu+loco
Stress/Metal

Supplementary Fig. 8: SDNs of malignant cells.

(A) Distribution of the number of surrounding cells for individual malignant cells with different radii.

(B) Normalized mutual information (NMI) score between malignant cell clusters with different radii. p-values were calculated based on 1,000 permutations. **, p-value <0.01.

(C) UMAP of malignant cell clusters based on the approach in Fig. 3A.

(D) Bubble plot of the proportions of each cell state (column) as the surroundings of each malignant cell cluster in (C). Each row represents the surroundings of one malignant cell cluster. Color and dot size indicate proportions. Cell subtypes with a proportion ≥ 0.01 were shown in this figure.

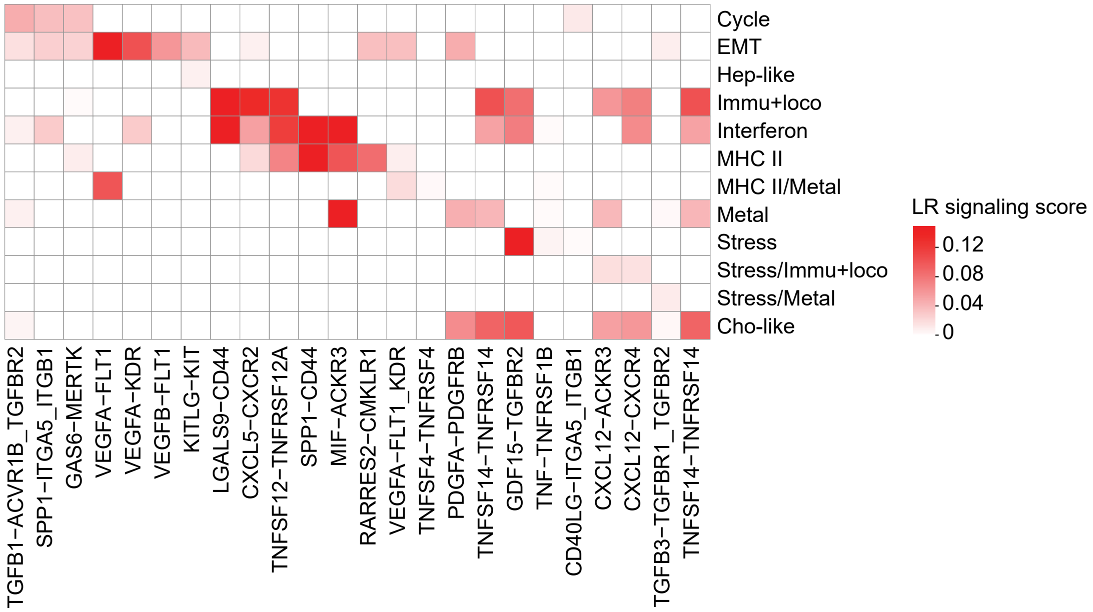

Supplementary Fig. 9: Crosstalk between malignant cell states and their local environments. Ligand-receptor interactions between each malignant cell state and their local environments.

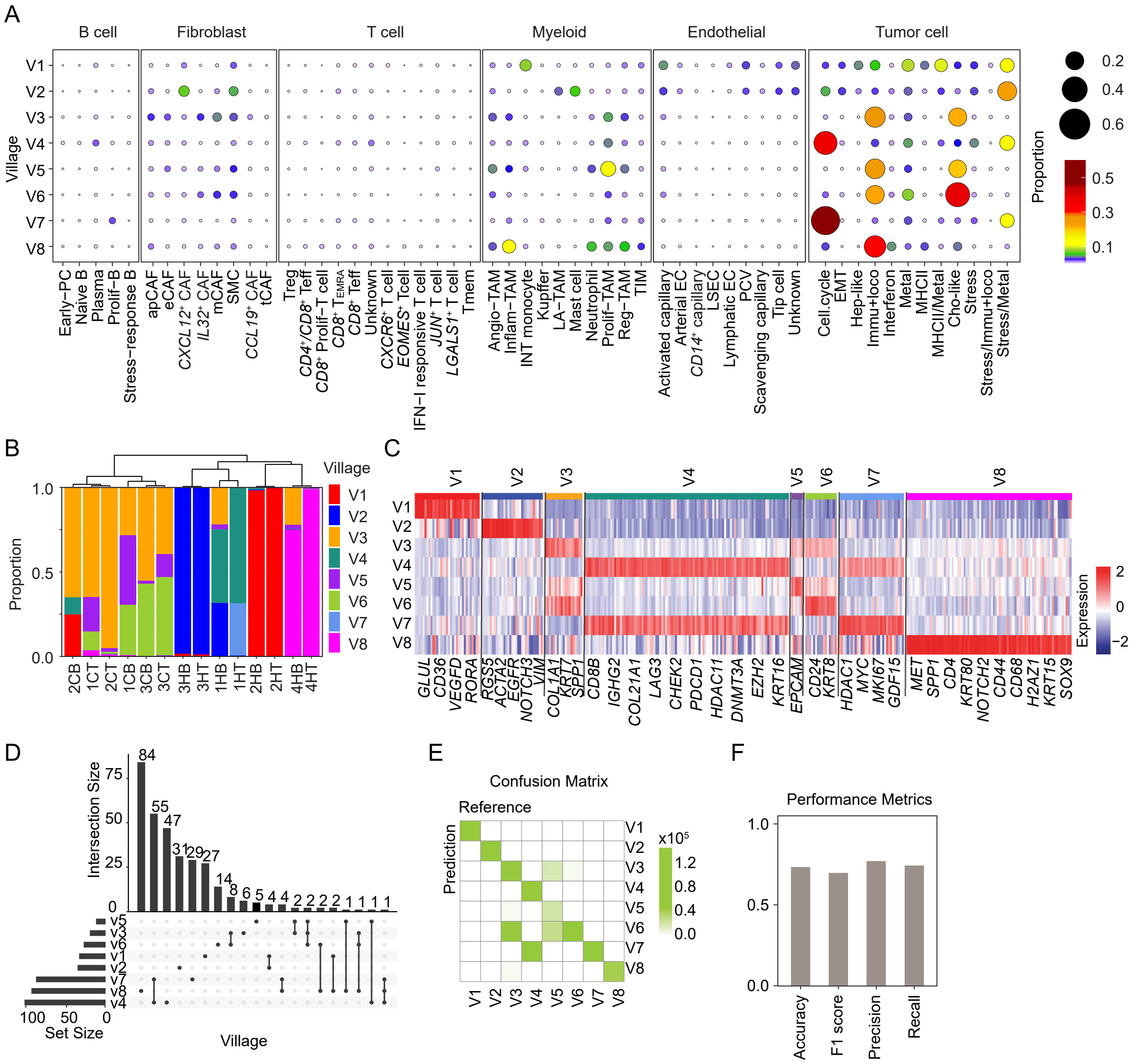
A
B cell
Fibroblast
T cell
Myeloid
Endothelial
Tumor cell
Village
V1
V2
V3
V4
V5
V6
V7
V8
Proportion
0.2
0.4
0.6
0.5
0.3
0.1
Early−PC
Naive B
Plasma
Prolif−B
Stress−response B
apCAF
eCAF
CXCL12+ CAF
IL32+ CAF
mCAF
SMC
CCL19+ CAF
tCAF
Treg
CD4+/CD8+ Teff
CD8+ Prolif−T cell
CD8+ TEMRA
CD8+ Teff
Unknown
CXCR6+ T cell
EOMES+ Tcell
IFN−I responsive T cell
JUN+ T cell
LGALS1+ T cell
Tmem
Angio−TAM
Inflam−TAM
INT monocyte
Kupffer
LA−TAM
Mast cell
Neutrophil
Prolif−TAM
Reg−TAM
TIM
Activated capillary
Arterial EC
CD14+ capillary
LSEC
Lymphatic EC
PCV
Scavenging capillary
Tip cell
Unknown
Cell.cycle
EMT
Hep-like
Immu+loco
Interferon
Metal
MHCII
MHCII/Metal
Cho-like
Stress
Stress/Immu+loco
Stress/Metal
B
Village
1.0
0.5
0.0
Proportion
2CB
1CT
2CT
1CB
3CB
3CT
3HB
3HT
1HB
1HT
2HB
2HT
4HB
4HT
C
Expression
2
0
−2
GLUL
CD36
VEGFD
RORA
RGS5
ACTA2
EGFR
NOTCH3
VIM
COL1A1
KRT7
SPP1
CD8B
IGHG2
COL21A1
LAG3
CHEK2
PDCD1
HDAC11
DNMT3A
EZH2
KRT16
EPCAM
CD24
KRT8
HDAC1
MYC
MKI67
GDF15
MET
SPP1
CD4
KRT80
NOTCH2
CD44
CD68
H2AZ1
KRT15
SOX9
D
Intersection Size
75
50
25
0
84
55
47
31
29
27
14
8
6
5
4
4
2
2
2
2
1
1
1
1
v5
v3
v6
v1
v2
v7
v8
v4
100
50
0
Set Size
Village
E
Confusion Matrix
Reference
Prediction
x10^5
1.2
0.8
0.4
0.0
F
Performance Metrics
1.0
0.5
0.0
Accuracy
F1 score
Precision
Recall

Supplementary Fig. 10: Features of tumor cell villages.

(A) Bubble plot of the compositions of different cells in each tumor village. Color and dot size indicate proportions.

(B) Proportions of tumor cell villages in each sample. Sample IDs were named based on histological subtypes of liver cancer, where H represents HCC and C represents iCCA.

(C) Heatmap of the differentially expressed genes of each tumor village. Representative markers were indicated.

(D) Upset plot of the overlap of village-specific genes. Left: bar plot of the total number of genes (x axis) for each village (y axis). Right: intersection of genes among different villages. Each column represents a set of genes either unique to each village (dark dots) or shared between villages (connected dots). The number of genes in each set is indicated as the height of the bar with a number, while sets of shared genes are indicated using dots, with villages indicated on the left.

(E) Confusion matrix between the determined tumor villages using graph attention networks and those predicted based on village-specific marker genes.

(F) Performance of village prediction by village specific marker genes.

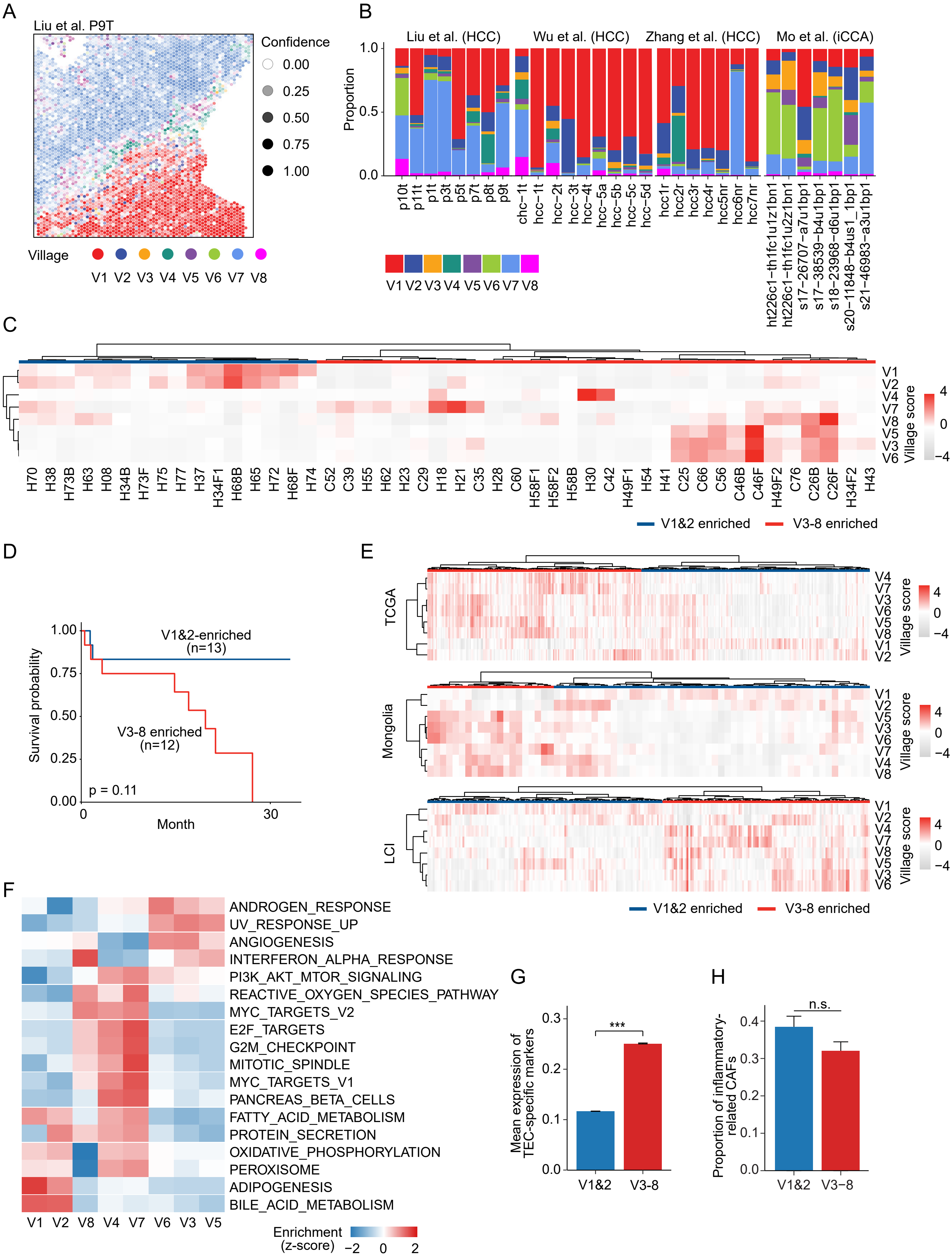
A
Liu et al. P9T
Confidence
0.00
0.25
0.50
0.75
1.00
Village
V1 V2 V3 V4 V5 V6 V7 V8
B
Liu et al. (HCC)
Wu et al. (HCC)
Zhang et al. (HCC)
Mo et al. (iCCA)
Proportion
1.0
0.5
0.0
p10t p11t p1t p3t p5t p7t p8t p9t
chc−1t hcc−1t hcc−2t hcc−3t hcc−4t hcc−5a hcc−5b hcc−5c hcc−5d
hcc1r hcc2r hcc3r hcc4r hcc5nr hcc6nr hcc7nr
ht226c1−th1fc1u1z1bn1 ht226c1−th1fc1u2z1bn1 s17−26707−a7u1bp1 s17−38539−b4u1bp1 s18−23968−d6u1bp1 s20−11848−b4us1_1bp1 s21−46983−a3u1bp1
V1 V2 V3 V4 V5 V6 V7 V8
C
V1 V2 V4 V7 V8 V5 V3 V6
Village score
4
0
−4
H70 H38 H73B H63 H08 H34B H73F H75 H77 H37 H34F1 H68B H65 H72 H68F H74 C52 C39 H55 H62 H23 C29 H18 H21 C35 H28 C60 H58F1 H58F2 H58B H30 C42 H49F1 H54 H41 C25 C66 C56 C46B C46F H49F2 C76 C26B C26F H34F2 H43
V1&2 enriched
V3-8 enriched
D
Survival probability
1.00
0.75
0.50
0.25
0.00
V1&2-enriched
(n=13)
V3-8 enriched
(n=12)
p = 0.11
0
30
Month
E
TCGA
V4 V7 V3 V6 V5 V8 V1 V2
Mongolia
V1 V2 V5 V3 V6 V7 V4 V8
LCI
V1 V2 V4 V7 V8 V5 V3 V6
Village score
4
0
−4
V1&2 enriched
V3-8 enriched
F
ANDROGEN_RESPONSE
UV_RESPONSE_UP
ANGIOGENESIS
INTERFERON_ALPHA_RESPONSE
PI3K_AKT_MTOR_SIGNALING
REACTIVE_OXYGEN_SPECIES_PATHWAY
MYC_TARGETS_V2
E2F_TARGETS
G2M_CHECKPOINT
MITOTIC_SPINDLE
MYC_TARGETS_V1
PANCREAS_BETA_CELLS
FATTY_ACID_METABOLISM
PROTEIN_SECRETION
OXIDATIVE_PHOSPHORYLATION
PEROXISOME
ADIPOGENESIS
BILE_ACID_METABOLISM
V1 V2 V8 V4 V7 V6 V3 V5
Enrichment
(z-score)
−2 0 2
G
Mean expression of
TEC-specific markers
0.3
0.2
0.1
0.0
***
V1&2
V3-8
H
Proportion of inflammatory-
related CAFs
0.4
0.3
0.2
0.1
0.0
n.s.
V1&2
V3−8

Supplementary Fig. 11: Validation of tumor cell villages using independent datasets.

(A) A representative example of tumor cell villages in Liu et al. The alpha levels of the spot indicate the confidence of village prediction.

(B) Proportions of tumor cell villages in each tumor sample from four publicly available 10X Visium datasets.

(C) Hierarchical clustering of tumor samples from a single-cell cohort (Ma et al.) based on village-specific surrogate marker genes. Sample IDs were named based on histological subtypes of liver cancer, where H represents HCC and C represents iCCA.

(D) Overall survival of HCC patients enriched for V1&2 related features (blue) and V3-8 related features (red) from the single-cell cohort (Ma et al.). p-value was calculated using the log rank test.

(E) Hierarchical clustering of tumor samples in TCGA, Mongolia, and LCI cohorts based on village scores. Village score was determined as average expression of village-specific surrogate marker genes.

(F) Enriched pathways of different tumor cell villages. For each identified pathway, enrichment scores were further scaled (z-score transformation) across tumor cell villages.

(G) Averaged expression of TEC-specific genes in endothelial cells from V1&2 compared with V3-8. Mean + SEM of the values are shown. p-value was calculated using one-sided Student's t-test. ***, p-value <0.001. Source data are provided as a Source Data file.

(H) Proportions of inflammatory-related CAFs (*IL32*+ CAFs, *CCL19*+ CAFs, and *CXCL12*+ CAFs) in V1&2 compared with V3-8. Mean + SEM of the values are shown. P-values were calculated using two-sided Student's t-test. n.s., not significant. Source data are provided as a Source Data file.

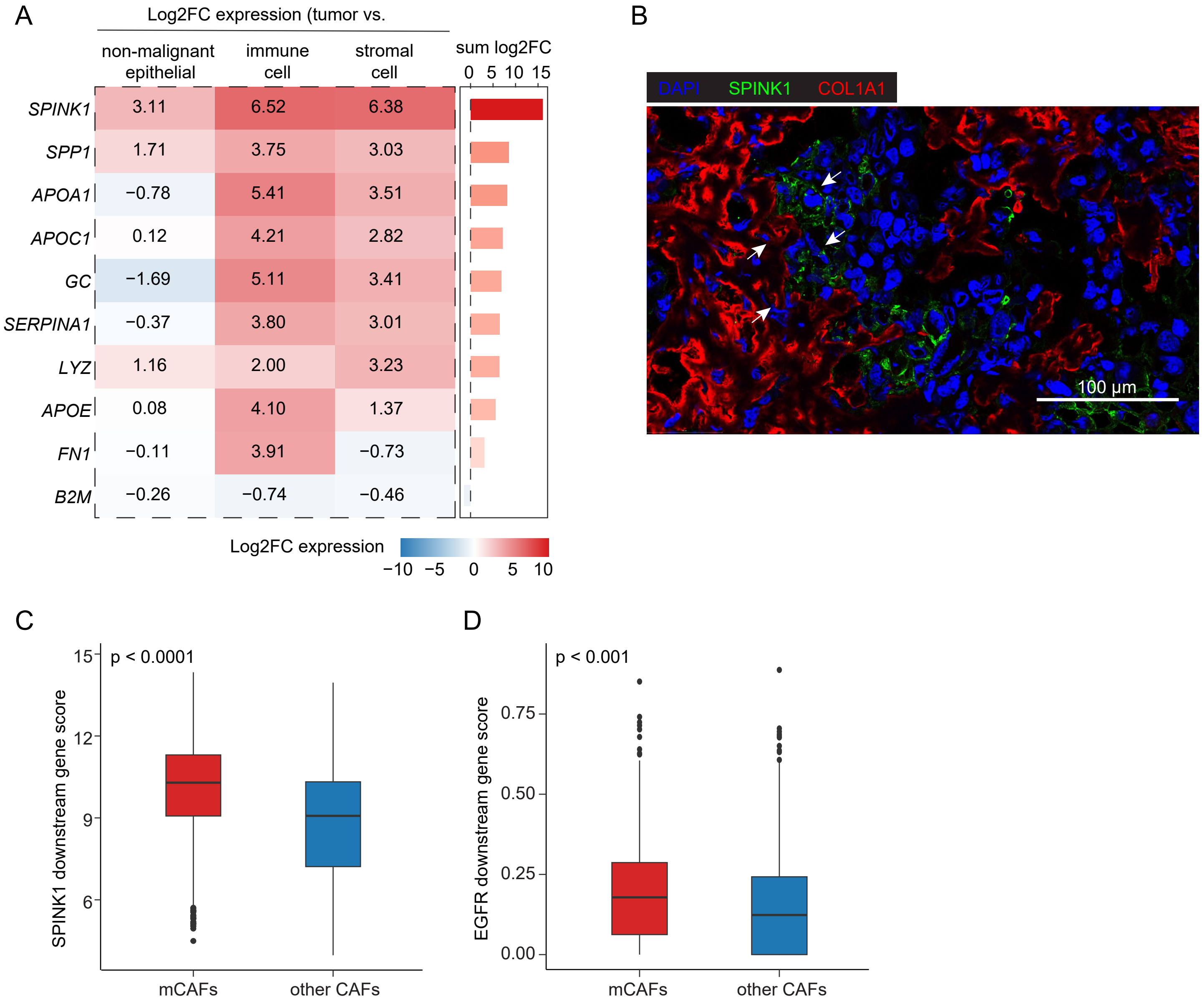

Supplementary Fig. 12: Spatial molecular co-dependencies in individual tumor cell villages.

(A) Log2 foldchange (log2FC) of the expression of genes (row) in tumor cells compared with other cell types (column). Color indicates log2FC level. Right panel showing summed value of log2FC for each gene across columns.

(B) Multiplex immunofluorescence staining of SPINK1 (green) and COL1A1 (red) for patient 3C. Nuclei staining with DAPI is indicated in blue. The staining was performed one time. Scale bar, 100 μm.

(C-D) Downstream gene score of SPINK1 (C) and EGFR (D) in mCAFs and other CAFs. Each box shows the median (center line), interquartile range (box), and data range (whiskers). Downstream genes of SPINK1 were obtained from the NicheNet database. EGFR downstream genes of PIK3CA, PIK3R1, KRAS, NRAS, HRAS, GRB2, SOS1 were used. Statistical significance was determined using one-sided Student's t-test. Source data are provided as a Source Data file.
