# Supplementary material for: Tumor cell villages define the co-dependency of tumor and microenvironment in liver cancer
Source: Nat Commun. 2026 Feb 21;17:1986. doi: 10.1038/s41467-026-69797-z (PMC12932787; doi:10.1038/s41467-026-69797-z)
Supplement: Supplementary file 2 — Description of Additional Supplementary Files [file 41467_2026_69797_MOESM2_ESM.pdf]

Description of Additional Supplementary Files

Supplementary Data 1: Clinical and sample information.

Supplementary Data 2: Genes for the annotation of major cell types.

Supplementary Data 3: Genes in different modules.

Supplementary Data 4: Number of cells in each tumor cell state for individual samples.

Supplementary Data 5: Gene signatures of each tumor cell village.

Supplementary Data 6: Top gene pairs in each tumor cell village.

Supplementary Data 7: Ligand-receptor interactions between SPINK1+ tumor cells (senders) and CAFs (receivers).

Supplementary Data 8: Ligand-receptor interactions between SPINK1+ tumor cells (receivers) and CAFs (senders).
